# Evaluating qualitative data analysis workshops from the perspective of public contributors

**DOI:** 10.1186/s40900-024-00628-5

**Published:** 2024-09-27

**Authors:** Alice Moult, Carmel McGrath, Kate Lippiett, Caroline Coope, Andrew Turner, Simon Chillcott, Linda Parton, Pam Holloway, Sally Dace, Andy Gibson, Clare Jinks, Zoe Paskins, Mari Carmen Portillo, Cindy Mann, Krysia Dziedzic

**Affiliations:** 1 Impact Accelerator Unit, Keele University, Newcastle-under-Lyme, ST5 5BG UK; 2 NIHR Health Protection Research Unit in Behavioural Science and Evaluation, Population Health Sciences, Bristol Medical School, University of Bristol, Bristol, UK; 3 The National Institute for Health and Care Research Applied Research Collaboration West (NIHR ARC West) at University Hospitals Bristol and Weston NHS Foundation Trust, Bristol, UK; 4 Faculty of Health and Applied Sciences, School of Health and Social Wellbeing, University of West England, Bristol, UK; 5 School of Health Sciences, NIHR ARC Wessex, University of Southampton, Southampton, UK; 6 Centre for Academic Primary Care, Bristol Medical School (Population Health Sciences), University of Bristol, Canynge Hall, 39 Whatley Road, Bristol, BS8 2PS UK; 7 National Institute for Health Research Applied Research Collaboration West (NIHR ARC West), University Hospitals Bristol and Weston NHS Foundation Trust, Bristol, BS1 2NT UK; 8 Centre for Academic Primary Care, University of Bristol, Bristol, BS8 2PS UK; 9 Centre for Musculoskeletal Health Research and Keele Medical School, Keele University, Keele, Staffordshire ST5 5BG UK; 10 Haywood Centre for Academic Rheumatology, Midlands Partnership University NHS Foundation Trust, Stoke on Trent, ST6 7AG UK; 11 NIHR ARC Wessex, School of Health Sciences, University of Southampton, Southampton, SO171JB UK

**Keywords:** Patient and public involvement, Evaluation, Qualitative research, Cube

## Abstract

**Background:**

The aim of this project is to evaluate public contributors’ experiences of their involvement in qualitative data analysis workshops during an on-going research project titled ‘Personalised Primary care for Patients with Multiple long-term conditions’.

**Methods:**

Four qualitative data analysis workshops were designed and conducted between August and December 2023. We used the Cube evaluation framework (henceforth referred to as the Cube) to evaluate the workshops. The Cube suggests four domains for successful PPI (voice, agenda, change, contribute).Within Workshops One, Two and Three public contributors had to login to an account to access the Cube; this was modified in Workshop Four following feedback from public contributors.

**Findings:**

Across the four workshops the Cube was completed 11 times. Across all four workshops, public contributors thought that their voice was heard, that there were diverse ways to contribute and that they led the agenda. Public contributors thought that researchers responded to their questions and issues, when necessary.

**Conclusion:**

This evaluation has shown that public contributors can gain new skills and lead qualitative data analysis discussions.

**Supplementary Information:**

The online version contains supplementary material available at 10.1186/s40900-024-00628-5.

## Abstract (plain language summary)

Why are we doing this project?

Patient and public involvement (PPI) can enhance qualitative research methods, however, public contributors may need support in order to contribute. The aim of this project is to evaluate public contributors’ experiences of qualitative data analysis workshops during an on-going research project titled ‘Personalised Primary care for Patients with Multiple long-term conditions (PP4M)’.

How did we do this project?

Four qualitative data analysis workshops were co-designed and conducted between August and December 2023. The Cube evaluation framework (henceforth referred to as the Cube) was used to evaluate each workshop. The Cube suggests four domains for successful PPI (voice, agenda, change, contribute). Within Workshops One, Two and Three public contributors had to login to an account to access the Cube; this was modified in Workshop Four following feedback from public contributors.

What did we learn?

Across the four workshops the Cube was completed 11 times. Public contributors thought that their voice was heard, that there were diverse ways to contribute and that they led the agenda. Public contributors thought that researchers responded to their questions and issues, when necessary. This evaluation has shown that public contributors can gain new skills and lead qualitative data analysis discussions.

**Supplementary Information:**

The online version contains supplementary material available at 10.1186/s40900-024-00628-5.

## Background

Patient and Public Involvement (PPI) in research can be defined as research carried out “with” or “by” patients and public contributors rather than “to”, “about” or “for” them [1]. Various justifications from doing public involvement in research have been articulated, including both rights-based approaches and more instrumental concerns regarding improving the quality of health research [2]. Underpinning all these justifications is an opposition to both medical and academic paternalism, aptly summed up in the dictum, “Nothing about us, without us” [3]. This motto has its origins outside of the United Kingdom (UK) and was first widely used in the English context as part of disability rights activism in the 1990s [4]. Since then its use has spread into many other areas [3].

PPI is put into practice through people with lived experience of health issues or healthcare discussing, helping to make decisions and conducting research to enhance study relevance, design, recruitment, data analysis, reporting and governance [5–7]. Crucially the promise that health research will be of benefit to patients, carers and the public cannot be taken as read [9]. Political, economic and cultural factors influence what research gets done, by whom and whether the findings are implemented [10]. Public involvement can be seen as one of the processes that helps to ensure that the promise to improve people’s health and wellbeing through research is delivered on [2]. Within the UK, the National Institute for Health and Care Research (NIHR) is the major funding body for health research and requires researchers, when applying for funding, to describe how they have involved the public in the design and planning of the project as well as plans for further involvement throughout the study [11].

Involving public contributors can enhance qualitative research methods [12]. For example, public contributors can add to the credibility of qualitative research by ensuring lived experience perspectives inform data analysis [12]. People with lived experience of the issues involved will bring their own knowledge to bare on the data, potentially identifying different issues and interpretations from those identified by academics. When integrating PPI into any phase of a study it is important to consider the skills required of public contributors for meaningful involvement and their further development. One of the six UK Standards for Public Involvement, designed to improve the quality and consistency of PPI in research, is ‘Support and Learning.’ This standard suggests that researchers should offer learning opportunities for public contributors to build their skills [13]. Providing or offering training and support ensures reciprocal learning opportunities and may help public contributors to feel confident when accessing and engaging in PPI activities [14].

Concurrent with the rise of PPI in research is a requirement for greater evaluation of that PPI, to determine its value and quality [15]. The process of PPI evaluation allows public contributors to consider whether their participation has been meaningful and is encouraged by the UK Standards for Public Involvement through the standard ‘Impact’ [13]. A range of methods are available to evaluate PPI in research, often chosen based on the intended outcomes of the research and the time frame and resources available. These approaches range in simplicity, from preparing an ‘impact log’ on the outcomes of the PPI [16], using the Cube evaluation framework (henceforth referred to as the Cube) [17], to the more comprehensive and resource intensive Public Involvement Impact Assessment Framework [18] or a Realist Evaluation [19].

The Cube was developed to provide a relatively sophisticated approach to evaluation, while minimizing the burden placed on researchers and public contributors. It was developed through a combination of reviewing the theoretical literature on social inequality and practical workshops with public contributors [17]. Creating the conditions for equitable knowledge exchange between public contributors’ and researchers can only be achieved if certain conditions in the research process are met. The Cube was developed to provide some clarity to what these conditions might be. The framework describes four fundamental domains for successful knowledge exchange. In combination these domain allow for the dynamic, fluid and sometimes unpredictable nature of interactions within knowledge spaces. The four domains are: voice (the extent to which contributors feel they have a weak or strong voice in decision-making); contribute (the number of ways to get involved to accommodate different contributors' needs); agenda (the balance between organisation and public contributor concerns); change (the willingness or resistance to change by the organisation or project). It can be used to compare public contributors’ experiences of PPI across different organisations or across time within a project or workstream, as in this paper. Since the original publication of the Cube, an online version has been developed [17], allowing for greater flexibility with both remote and asynchronous input.

The aim of this paper is to evaluate the public contributors’ experiences of their involvement in qualitative data analysis workshops during an on-going research project titled ‘Personalised Primary care for Patients with Multiple long-term conditions (PP4M)’. We will do this through reporting results from using the Cube to evaluate a series of qualitative data analysis workshops, held for public contributors, which were embedded throughout an on-going research project titled ‘Personalised Primary care for Patients with Multiple long-term conditions (PP4M)’.

### Personalised primary care for patients with multiple long-term conditions (PP4M)

The PP4M study aimed to support the implementation of a smart template for use by primary care staff to promote personalised care for patients with multiple long-term conditions. The PP4M study investigated barriers and facilitators of implementation, and evidence of impact in meeting the aim of providing more personalised care. The PP4M study was conducted across three locations in the UK; Bristol, Keele (Stoke-on-Trent) and Southampton. The study was a collaboration between four NIHR Applied Research Collaborations (ARCs): ARC West, West Midlands, Wessex, and South West Peninsula.

The scope of this PPI evaluation focuses on the activities that contributed to the qualitative data analysis within the PP4M study (PP4M involved a range of qualitative and quantitative methods). For the PP4M study, qualitative data was collected from participating general practices from the three locations. Researchers conducted interviews with patients and staff members. Patients were interviewed about their experience of care in general practice for their long-term health conditions, and about their experience of their consultations after the template had been introduced. Clinical staff were interviewed about their experiences implementing and using the template. Analysis of this qualitative data set was the subject of the PPI data analysis workshops which were evaluated.

We now describe prior PPI activities within PP4M, the methods used in this project including design and overview, identifying public contributors, ethical considerations, planning of workshops, workshop content, data analysis and results.

### Prior PPI activities

Public contributors were key members of the research team and, at the very start of the study, co-developed a PPI plan outlining the various ways in which they wanted to contribute throughout the study. Alongside the PPI plan, public contributors also provided ad hoc input on issues that arose during the study, for example, on recruitment of patients which proved challenging. The proposed PPI activities relating to qualitative data collection and analysis have previously been published [20].

A summary of our PPI activities within this evaluation are reported using the GRIPP 2 Short Form in Supplementary Material 1.

## Methods

### Design and overview

Four workshops were delivered to support public contributors’ involvement in qualitative data analysis. The Cube was used to evaluate each workshop.

### Identifying public contributors

Public contributors had lived experiences of multiple long-term conditions or had recent experiences of primary care services. Some public contributors had been involved in qualitative research projects before, whilst others had not. The recruitment of public contributors into the local PPI groups differed in each region. Members of the Keele PPI group were recruited through an existing Research User Group (RUG) hosted by the University’s Impact Accelerator Unit. To ensure diversity within the RUG a Race Equality Ambassador works with underserved groups with the vision to invite them to be part of the RUG. In Southampton, public contributors were recruited through the NIHR ARC Wessex PPI group. In Bristol an advertisement was sent out through local public involvement mailing lists including the People in the Health West of England. This network is a regional collaboration led by the University of the West of England bringing together key research partners and public contributors from across the NIHR and beyond to work jointly on public involvement. CMcG reviewed the responses, seeking to recruit individuals with diverse backgrounds and lower socio-economic status who had provided a brief self-description and reasons for wanting to be involved.

### Ethical considerations

Following joint guidance from the National Research Ethics Service and the Health Research Authority [21], ethical approval was not sought for this project. Active involvement in PPI and its evaluation was conducted with the contributors as equal partners, rather than research participants. The workshops were conducted with the utmost respect and care for public contributors, allowing contributors to share the details they chose to during the workshops. All contributors gave permission for the workshops to be recorded.

### Planning

Given the distance between locations, workshops were conducted online via Zoom. To create inclusive and accessible opportunities, public contributors were given the option to meet with a member of their local academic research team in person, in addition to joining the main group via Zoom. Workshops were sometimes held on multiple occasions.

To plan the workshops, public contributors were asked to complete a short Microsoft Form asking their preference on the type of qualitative content (e.g. more theoretically driven or with practical elements), structure of the workshop, time of day and platform they would like the workshop to be hosted on (please see Supplementary Material 2 for a list of preference questions). The results from the responses provided the foundations for the first and second workshops. The project team and public contributors from all three regions also met via Zoom to discuss the content of Workshop One. Originally, Workshop One intended to cover the theory behind the approach to qualitative analysis as well as a practical activity. However, it was decided that more time should be provided for asking questions, therefore introducing public contributors to qualitative methods was delivered over two workshops; one which focused on theory and the other focusing on practical examples. Workshops Two, Three and Four were developed based on the responses from the Cube and meetings with public contributors.

### Workshop content

**Fig. 1:**
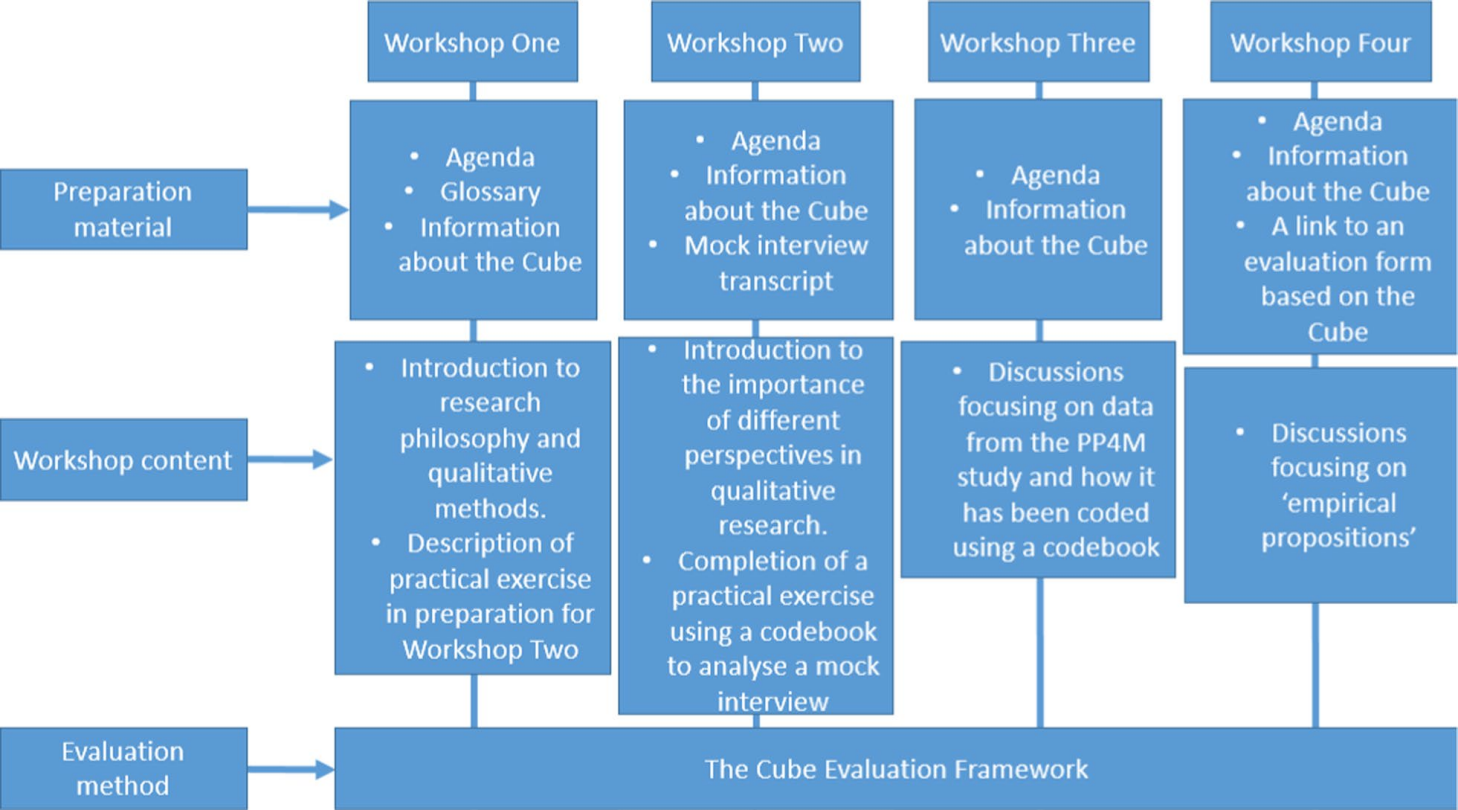
Description of workshops

Figure 1 depicts the preparation materials, content and evaluation methods of each workshop.

Workshop One: “An introduction to qualitative data analysis” – Part One

The first workshop aimed to provide an introduction to qualitative data analysis and was delivered by CMcG, KL, AT and AM. Prior to the workshop, the public contributors were emailed an agenda, a glossary explaining the terms that would be used during the workshop (please see Supplementary Material 3) and information about the Cube. Workshop One was broad in nature and included time at the start for the group to meet one another and share what they would like to get out of the training. The next section of the workshop included a brief description of the study’s philosophical positioning, the methodology (qualitative research) [22], and analysis methods (thematic [23] and framework [24]). During the explanations, public contributors were invited to ask questions which shaped the content covered during the workshop. Public contributors were offered a 15-min break. Public contributors were then introduced to the practical data analysis activity that they would be completing Workshop Two.

The workshop ended with CMcG explaining plans for next steps and the public contributors logging into an account to access and complete the Cube, led by AG. An independent researcher (AG) facilitated the Cube evaluation to encourage more candid feedback. The scores and descriptions from the Cube were collected and discussed prior to the workshop ending.

The workshop was recorded, edited and re-sent to the public contributors as a reminder ahead of Workshop Two which was held at a later date.

Workshop two: “An introduction to qualitative data analysis – Part Two”

The second workshop aimed to build upon Workshop One and to have a practical data analysis exercise. The workshop was delivered by CMcG, KL, AT and AM. An agenda and information describing the Cube was sent to all public contributors prior to the workshop. The workshop began with an exercise which involved the public contributors sorting out a pack of cards and explaining why they sorted the cards in that particular way [25]. The idea of the exercise was to introduce the concept of perspectives, illustrating how, in qualitative research, data can be made sense of in different ways, depending on one’s own perspective and rationale, reinforcing the importance of reflexivity and open discussion in qualitative research. The next activity involved a practical exercise to demonstrate the processes used by researchers when coding data. This activity was intended to (1) illustrate what happens ‘behind the scenes’ when researchers are undertaking qualitative data analysis, (2) provide a fun and engaging learning experience, and (3) at later stages within the workshop, generate in-depth discussions about the findings from the analysis. Prior to the second workshop, public contributors were either emailed or posted an interview transcript. The transcript was derived from a mock interview conducted by SC (public contributor playing the role of a nurse) who interviewed CM (role playing as a patient), from a topic guide developed from the study topic guides This was to generate material to reflect the type of data the researchers would be analysing, without using real study data.

Public contributors were then introduced to Normalisation Process Theory (NPT) [26], which was chosen as the main framework of this study because it aims to understand the implementation processes by which complex interventions (e.g. the new smart template) are operationalised and sustained in practice. Each public contributor worked with a researcher to code the mock transcript using a codebook derived and adapted from NPT [26], again to replicate the type of coding framework that would be used by the researchers working on the study. Following the activity, there was space in the workshop for the group (facilitated by CMcG) to spend time discussing how and what they had coded to each NPT domain.

The workshop concluded with the public contributors logging into an account to access and complete the Cube, led by AG. The scores and descriptions from the Cube were collected and discussed prior to the workshop ending. This workshop was held twice to permit public contributors from each region to attend.

Workshop three: “Helping the researcher to interpret the data”

This workshop aimed to use the thematic codebook approach to data analysis [27] and was delivered by CMcG, KL, AT and CC. An agenda and description of the Cube was sent to all public contributors prior to the workshop.

The timing of this workshop had enabled early input from the PPI members on the initial coding and analysis. At the start of the workshop, CC explained the thematic codebook approach and provided time for the attendees to ask questions. CC then presented the codebook generated from the data, with illustrative quotes provided for additional context. Following public contributor feedback from the earlier workshops, the group were provided with a longer period of time to discuss and comment on the interpretations of the data.

The workshop concluded with public contributors logging into an account to access and complete the Cube, led by AG. The scores and descriptions were collected and discussed prior to the workshop ending.

Workshop Four: “Helping the researcher to interpret the data”

The final workshop aimed to provide public contributors the chance to discuss the key findings and was delivered by CMcG, CC and KL. An agenda was circulated ahead of the workshop including a link to the evaluation form; this form replicated the Cube without the public contributors having to login to an account to access the evaluation. This workshop was held at a later stage of the analysis. Public contributors were given a number of empirical propositions (statements that explained key elements of the data and were used in the development of themes) with associated verbatim quotes. KL invited public contributors to choose the empirical propositions that interested them most and encouraged open discussion.

Following feedback from public contributors, the majority of the workshop was allocated to group discussion, with each member bringing in their thoughts and perspectives on the data and key messages that were coming through. Following the workshop, the public contributors were asked to complete a short Microsoft Office form including the four questions relating to each Cube domain and free-text boxes underneath each question for any other comments.

### Follow-up sessions

Following the workshops, informal ‘drop-in’ sessions were organised (1 h per week for 3 weeks) to create space for researchers and public contributors to talk about progress with the qualitative data analysis. Researchers reflected on how they had learnt how to involve public contributors in qualitative data analysis and how the transparency of the analysis process and richness of discussions enhanced the interpretation of the data. The Cube was not discussed in these sessions, nor was it used to evaluate these sessions.

### The Cube framework

The Cube [16] was chosen for this project to reflect the consideration of the differing ‘knowledge spaces’ (the conversation space in which different types of expertise from the public and researchers are shared) that are important when evaluating interactions between the public and academics on healthcare issues.

For Workshops One, Two and Three, public contributors were asked to complete the online version of the Cube. Each public contributor registered for an account and had to log onto the online platform. Four questions, one relating to each domain of the Cube, were adapted for this project (Table 1). Public contributors answered each of the questions by using a slider under the question from 0 to 1 on each dimension. This creates a data point on the Cube, which moves in three dimensions and changes colour according to the final question on ‘change’. A comment box appeared for each question so that public contributors could add additional details on their experience in relation to each domain. The scores and comments were recorded in a spreadsheet associated with the Cube.

**Table 1:** The four dimensions of the ‘Cube’

Dimension	Description	Questions asked within the workshops
Voice	Strong voices discuss issues and influence decision-making. Weak voices may discuss issues but have little influence on decision-making	Were you able to ask questions and influence the introductory workshops?
Contribute	Knowledge can take on different forms, which may not be equally valued. A single involvement approach is likely to privilege one social/cultural group over another, thus perpetuating inequality	Were there different ways to be involved in the introductory workshops (online, in-person)?
Agenda	Public concerns are the issues and topics that matter to the patients and public involved. These may coincide or be distinct from the concerns that are most relevant to academic organisations	Was the content of the introductory workshops appropriate?
Change	Decision-makers’ willingness and ability to respond to issues raised by public contributors in knowledge spaces depend on contextual factors	Did the researchers respond to issues throughout the introductory workshops?

Following discussions around the logistical challenges of each public contributor having to log onto the online platform to access the Cube, for Workshop Four CMcG transported each question into a Microsoft Form. Public contributors were asked to rate each question on a Likert Scale. Contributors could add additional details on their experience in relation to each dimension) in free-text boxes under each question.

### Analysis

The scores for each workshop were collated and summarised by the median within each domain. Comments made by contributors were collated into a table to summarise their experiences of each workshop and narratively grouped into common themes.

## Results

Each workshop was approximately 2 h and 30 min in duration. Table 2 details the date of each workshop and the number of public contributors and staff who attended. All public contributors were White British.

**Table 2:** Workshop information

	Date of workshop	Number of public contributors who attended	Number of staff who attended
Workshop One	15th August 2023	7	5
Workshop Two	15th September 2023	4	5
Workshop Two	29th September 2023	2	5
Workshop Three	6th October 2023	4	6
Workshop Three	13th October 2023	2	4
Workshop Four	22nd November 2023	4	3

### Quantitative Cube results

Table 3 describes the median values for each Cube domain in each workshop and provides the median value of all four domains for each workshop.

**Table 3:** Quantitative Cube results

		Workshop One N = 2	Workshop Two N = 2	Workshop Three N = 3	Workshop Four N = 4	Score of all respondents N = 11
	Questions relating to each domain	Median score	Median score	Median score	Median score range	Median Score
Voice	Were you able to ask questions and influence the introductory workshops?	0.88 (0.85–0.91)	0.845 (0.8–0.89)	0.91 (0.81–0.92)	0.7 (0.6–0.7)	0.79
Contribute	Were there different ways to be involved in the introductory workshops (online, in-person)?	0.725 (0.59–0.86)	0.665 (0.64–0.69)	0.78 (0.66–0.9)	0.7 (0.2–0.8)	0.69
Agenda	Was the content of the introductory workshops appropriate?	0.85 (0.83–0.87)	0.695 (0.5–0.89)	0.86 (0.81–0.91)	0.7 (0.6–0.8)	0.78
Change	Did the researchers respond to issues throughout the introductory workshops?	0.87 (0.82–0.92)	0.25 (0.1–0.4)	0.83 (0.7–0.93)	0.7 (0.6–0.7153	0.67

N = the number of public contributors who complete the Cube

Score range (0–1) Higher is better; Scores 0.0–0.3 did not agree *Scores 0.31–0.6 somewhat agree *Scores 0.61–1.00 agree

Across all four workshops, public contributors thought that their voice was heard, that there were diverse ways to contribute and that they led the agenda. Responses to the domain ‘change’ varied more across the workshops with Workshop Two receiving the lowest score of 0.25.

### Free text responses

Public contributors valued having the choice to attend each workshop either in-person or via Zoom. Public contributors who attended in-person and online suggested that they were able to ask questions throughout the workshops and that researchers were responsive to their questions. Public contributors who attended Workshop Two suggested that they wanted this workshop to be led by the researchers as they have specialist knowledge and experience of qualitative methods; they did not want the content of the workshop to be changed in response to their questions which was why they scored the ‘Change’ dimension as the lowest.

In terms of preparation materials, a few of the public contributors who attended Workshop One and Three valued being sent information prior to the workshop as this enabled them to prepare and engage within the activities. Yet, both public contributors who attended Workshop Two suggested that whilst they appreciated being sent an interview transcript prior to the workshop, they did not know that information relating to the mock interview transcript would be explained to them within the workshop. This resulted in the public contributors spending potentially unnecessary time outside of the workshop trying to understand and analyse the interview transcript.

Within Workshop One a public contributor highlighted that prior discussions of the purpose and structure of the workshop ensured that the activities were relevant. Most public contributors commented on how the content from Workshop One flowed to Workshop Four. Two public contributors suggested that Workshop Four particularly brought together what they had learned in previous workshops. A few public contributors suggested that running Workshop Two and Three on multiple occasions enabled involvement from public contributors from each region.

All public contributors who attended Workshop Three described how they enjoyed interpreting data from the PP4M study. One public contributor suggested that they would have liked more time allocated within the workshop to discuss this. Two public contributors described how they felt like a researcher as their comments could influence how the data has been interpreted within the study. All public contributors reported how the workshops had helped demystify the research process and described feeling positive about the PP4M study and welcomed the use of the multi-morbidity template within general practice.

With regards to the Cube itself, one public contributor described that within Workshop Two, upon completion of the Cube activity, the scores and comments were shown to the group; this public contributor stated that their scoring of Workshop Three was influenced by this experience as they had made similar comments to the other public contributor in Workshop Two but their scoring was higher; the public contributor did not say in which way this influenced their scoring. All public contributors alluded to having issues when completing the Cube activity due to problems trying to login to the online platform, trying to understand the pragmatics of completing the activity remotely and loss of internet connection before completion of the activity within previous workshops.

## Discussion

The Cube was used to evaluate qualitative data analysis workshops, held across different locations, for public contributors, which were embedded throughout an on-going research project (PP4M). Overall, responses were positive to all four Cube domain across all four workshops. Public contributors felt there were diverse ways to contribute to the workshops, and that they had a strong voice to add to the discussion. Balance was achieved regarding whose concerns (public or researchers) led the agenda. Indeed, it was clear that, at times, public contributors thought it was important for researchers to lead the agenda (for example, when presenting on philosophical orientation and methodologies). However, contributors did also feel listened to—so they reported that researchers would make changes based on the discussion when it was necessary.

Previous literature has conceptualised ‘meaningful’ PPI as being based on the principles of valuing partnerships, cultivating learning and identifying and being responsive to training needs of public contributors [27]; these principles were the basis of designing and running the qualitative data analysis workshops. The implementation of the public contributors’ new research skills into practice was a key activity within the workshops and their new knowledge is a skill that each public contributor can take forward into future projects. Learning was reciprocal, so researchers also developed skills, for example teaching qualitative analysis to public contributors. Researchers welcomed the opportunity to step away from the ‘tunnel vision’ of data analysis into the stimulating and challenging yet ‘safe spaces’ of the workshops, which enhanced their own reflexivity in relation to the data; as discussed within the follow-up sessions.

Public contributors particularly valued having the opportunity to attend workshops either in-person or online. Some public contributors met with one of their local academic research team in person, in addition to joining the main group. This hybrid approach ensured a breadth of opinions and asynchronous discussion within and between public contributors from each region; thus showing that multi-site PPI groups can pragmatically work in practice. Despite this, workshop one was attended by 7 public contributors and, by the end of the project, workshop 4 was attended by 4 public contributors. This reduction in attendance could have been due to workshop 4 only being held once. In terms of preparation materials, public contributors valued being sent the resources, yet the purpose of these materials need to be clearly explained to mitigate any misunderstandings.

### Evaluating PPI

The benefits of using the Cube were that it enabled cross-sectional comparisons between the workshops and the results were immediately available allowing for activities to be modified in a timely manner. Whilst not directly explored, the lack of responses in the first two workshops could have been because due to logistical problems trying to logon to the online platform, trying to understand the pragmatics of completing the activity remotely and loss of internet connection before completing the evaluation which meant that results were lost. In particular, the time taken to log-on and access the evaluation could have been spent on data analysis and this was frustrating for researchers and public contributors. Once the researchers were aware of the logistical issues, they modified how the Cube was presented and all public contributors who attended Workshop Four completed the evaluation.

Evaluation frameworks for PPI have been criticised because the methods give precedence to indicators that might matter to researchers, not the public [28]. These frameworks often examine a one-way exchange of information that does not capture the reciprocal learning between researchers and the public. Staley and Barron’s [29] conceptualised PPI as conversations between public contributors and researchers which support mutual learning. Whilst the Cube only captured public contributor’s responses within this evaluation, it did prompt researchers to reflect upon their own PPI practices and to discuss these within the follow-up sessions. Completing the Cube activity is relatively quick in comparison to the Public Involvement Impact Assessment Framework [18] or conducting a realist evaluation [19], yet more time should have been allocated to the evaluation process within the workshops; this may have allowed for a greater discussion between the researchers and public contributors.

Primarily the evaluation of PPI activities is completed at the end of a project or study [30]. By evaluating and sharing learning from each workshop (e.g. that logging onto the online platform to complete the Cube activity was not practical), it maximised the impact on shaping the delivery of the subsequent workshops. Yet, by sharing the public contributors’ scores and comments within the group it did lead to some public contributors adjusting their subsequent scores for the following workshop to seemingly ‘match’ how the other public contributors scored; researchers may wish to be mindful of this when using the Cube to evaluate future PPI activities.

Studies which have asked public contributors to write narrative feedback when completing the Cube suggested that it encouraged contributors to reflect on their involvement and experiences [16, 31, 32], something which public contributors did in this evaluation. The narrative feedback helped to explain some of the scores, for example, within Workshop Two the domain ‘change’ was scored low. Public contributors suggested that they wanted Workshop Two to be led by the researchers and not changed as a response to public contributors’ opinions as it was the researchers who had expertise in qualitative methods. Whilst a comment box appeared for after each domain’s question, the narrative feedback written by public contributors did not always relate to that specific domain, for example, public contributors used this space to comment upon feeling positive about the PP4M study and welcomed the use of the multi-morbidity template within general practice. This illustrates how the Cube approach helps to generate discussion on the subtleties of PPI, rather than trying to evaluate PPI processes within a linear fashion.

### Strengths and limitations

This evaluation has shown the benefits of qualitative data analysis workshops for public contributors, however, one limitation may be that the presence of the researchers (either in-person or on the Zoom call) when completing the Cube activity may have influenced scores and feedback. To mitigate against this, the Cube activities within Workshops One, Two and Three were facilitated by AG, who was independent from the PP4M study. A further limitation of the evaluation was that all public contributors were over 50 years of age and White; more work is needed to ensure representation of under-served groups. All of the public contributors had experience of being involved in PPI activities which may have made it easier for them to contribute; public contributors with no prior experience may have needed more support in order to contribute. The small number of respondents to the Cube may limit transferability of learnings. Furthermore, given that public contributors bring their expertise through experience rather than in data analysis, it is important to bear in mind the potential tension of ‘teaching’ public contributors how to ‘do’ qualitative analysis.

### Recommendations for PPI within qualitative data analysis

From the learning gained from evaluating public contributors’ experiences of the qualitative data analysis workshops, we have co-produced several recommendations:

1. Identify learning needs of public contributors and co-produce resources or workshops that will address these learning needs
2. Before any PPI activity, send out relevant information sources or materials but ensure that there are clear goals and/ or instructions
3. Consider which stakeholders are required to run the sessions (e.g. qualitative researchers, PPI co-ordinator)
4. To be responsive to the public contributors during workshops to ensure their interests are central; this may mean being flexible in terms of activities

## Conclusion

Across all four workshops, public contributors thought that their voice was heard, that there were diverse ways to contribute and that they led the agenda. Public contributors thought that researchers responded to their questions and issues, when necessary. Public contributors valued being sent preparation materials, yet the purpose of these resources need to be clearly explained to mitigate any misunderstandings.

## Supplementary Information

Supplementary Material 1.

Supplementary Material 2.

Supplementary Material 3.

## Data Availability

Not applicable

## Abbreviations

NIHR: National Institute for Health and Care Research
PP4M: Personalised Primary care for Patients with Multiple long-term conditions
PPI: Patient and Public Involvement
UK: United Kingdom
