# Supplementary material for: Evaluating qualitative data analysis workshops from the perspective of public contributors
Source: Res Involv Engagem. 2024 Sep 27;10:99. doi: 10.1186/s40900-024-00628-5 (PMC11429467; doi:10.1186/s40900-024-00628-5)
Supplement: Supplementary file 1 — Supplementary Material 1. [file 40900_2024_628_MOESM1_ESM.docx]

# Additional File One The GRIPP2 Short Form

| Section and topic | Item |
| --- | --- |
| 1: Aim  Report the aim | To present details on the processes and activities planned to integrate PPI into the qualitative research component of a mixed-methods, multi-site study evaluating the implementation of a smart template to promote personalised primary care for patients with multiple long-term conditions. |
| 2: Methods  Provide a clear description of the methods used for patient and public involvement (PPI) in the study | This proposal describes the processes and activities planned to integrate PPI into the development and piloting of qualitative data collection tools (topic guides for both practice staff and patients) and a tailored training package developed for PPI members incorporating broad concepts and specific methods of qualitative data analysis. |
| 3: Results  Outcomes—Report the results of PPI in the study, including both positive and negative outcomes | Outputs relating to PPI activity may include clear, concise and suitably worded topic guides for qualitative interviews. Piloting of the topic guides via pilot interviews will further develop researchers’ skills including sensitisation to the experiences of participants being interviewed. Working with PPI members when analysing the qualitative data aims to provide reciprocal learning opportunities and may contribute to improving the overall rigour of the data analysis. |
| 4: Discussion  Outcomes—Comment on the extent to which PPI influenced the study overall. Describe positive and negative effects | The intent of publishing proposed PPI activities within this project is to inform the future delivery of high quality PPI. By being open and transparent about PPI processes this article could help others planning similar activities. |
| 5: Reflections  Critical perspective—Comment critically on the study, reflecting on the things that went well and those that did not, so others can learn from this experience | Throughout the proposed PPI activities, researchers will be reflective on worked well and what could be improved. Researchers will regularly meet to discuss any issues and changes to the proposal. |
