# Supplementary material for: Evaluating qualitative data analysis workshops from the perspective of public contributors
Source: Res Involv Engagem. 2024 Sep 27;10:99. doi: 10.1186/s40900-024-00628-5 (PMC11429467; doi:10.1186/s40900-024-00628-5)
Supplement: Supplementary file 2 — Supplementary Material 2. [file 40900_2024_628_MOESM2_ESM.docx]

**Workshop preference questions**

Question 1: What information would you like us to cover in the first meeting with you?

Options:

- Introduction to the PP4M project and this phase of work
- Your role in this phase of work
- What to expect
- Schedule of events
- A brief introduction to qualitative research
- Difference between qualitative research and quantitative research
- An introduction to Thematic Analysis
- A worked example of using Thematic Analysis
- A practical exercise of using Thematic Analysis
- Other

Question 2: The first training workshop will run for an hour- what format would you prefer?

Options:

- 30 minutes presentation, 30 minutes practical tasks
- Small sections of information with space for question and answers after each section
- Pre-reading before the meeting and the main meeting to be mostly practical
- Other

Question 3: What platform would you prefer the meeting to be on?

Options:

- Zoom
- Microsoft Teams
- Hybrid approach (in person and online).
- Other

Question 4: What time of day would you prefer the training session

Options:

- Morning (9am-12pm)
- Lunchtime (12pm-3pm)
- Afternoon (3pm-5pm)
- Evening (5pm onwards)
- Other

Question 5: Please use the space below to let us know if there is anything you would like to add that will help us to make sure the training meets your needs and preferences. If you have any questions please contact me on: carmel.mcgrath@uwe.ac.uk.
