# Supplementary material for: Evaluating qualitative data analysis workshops from the perspective of public contributors
Source: Res Involv Engagem. 2024 Sep 27;10:99. doi: 10.1186/s40900-024-00628-5 (PMC11429467; doi:10.1186/s40900-024-00628-5)
Supplement: Supplementary file 3 — Supplementary Material 3. [file 40900_2024_628_MOESM3_ESM.docx]

**Glossary**

For further information about the terms used in the glossary read: [Research Onion - Explanation of the Concept](https://www.ukessays.com/essays/psychology/explanation-of-the-concept-of-research-onion-psychology-essay.php)

| **Term** | **Definition/ Description** |
| --- | --- |
| Approaches | Different ways of exploring a research question or topic area. |
| Archival research | The study of previously recorded data (secondary data) and materials, such as historical documents, archives, and records. |
| Case Study | An in-depth study of a single person, group, event, or organisation |
| Choices | The options available for making decisions in a particular situation. |
| Cross-sectional | A research design that compares different groups of people or populations at the same point in time or over a short period. |
| Data collection and analysis | The process of collecting information and understanding it to make conclusions that answer a research question. |
| Deductive | A research method that explores a known theory and tests whether the theory is valid in different contexts through observation and data analysis. |
| Ethnography | Ethnography involves observing people in their own environment to understand their experiences, perspectives, and everyday practices. This information can provide detailed insights into a particular context, group, or culture. |
| Experiment | Experimental study designs involve changing certain things to see how the changes affects the outcome. |
| Grounded Theory | A research approach where the theory is developed based on the data collected through systematic steps rather than starting with an existing theory |
| Inductive | A research method where observations and findings are used to develop a theory |
| Interpretivism | A research philosophy that emphasises the importance of understanding human experiences, perceptions, and meanings in order to understand and gain knowledge of reality. |
| Longitudinal | A research design that follows the same group of people or population over an extended period to understand changes over time |
| Mixed methods | The combination of quantitative and qualitative research methods used in a study. |
| Mono method | The use of only one research method in a study |
| Normalisation Process Theory (NPT) | Explaining how a change becomes normal practice in everyday work |
| Philosophies | A set of beliefs and values that guide the approaches used to understand reality and develop knowledge about a research question or topic area. |
| Pragmatism | A research philosophy that focuses on practical solutions to develop knowledge and theory. |
| Realism | A research philosophy that emphasises the importance of objective reality and the use of evidence to form conclusions. |
| Strategies | The plans or methods that are used to achieve a specific aim. |
| Survey | A research design that involves collecting data from a sample of people using questionnaires |
| Techniques and procedures | The specific methods and steps used in research |
| Time horizons | The duration of time over which a research project is conducted. |
